# Enlisting the power of the verb

**DOI:** 10.1007/s40037-015-0177-9

**Published:** 2015-04-09

**Authors:** Lorelei Lingard

**Affiliations:** Centre for Education Research & Innovation, Department of Medicine, Schulich School of Medicine and Dentistry- Western University, Health Sciences Addition, Room. 112, N6A 5C1 London, ON Canada

In the writer’s craft section we offer simple tips to improve your writing in one of three areas: Energy, Clarity and Persuasiveness. Each entry focuses on a key writing feature or strategy, illustrates how it commonly goes wrong, teaches the grammatical underpinnings necessary to understand it and offers suggestions to wield it effectively. We encourage readers to share comments on or suggestions for this section on Twitter, using the hashtag: #how’syourwriting?

Verbs are the engine of a sentence: they propel the prose forward and infuse it with energy. Unfortunately, energy is precisely what much scholarly writing lacks. This problem can be attributed in part to ‘limp’ verbs and overuse of the passive voice. Consider this example:

*The literature* ***shows*** *that professionalism in medical students* ***has been discussed*** *in medical education from multiple perspectives.*

Two problems drain the energy from this sentence. First, verbs such as ‘shows’, ‘reports’, ‘does’ and ‘is’ (with its variants, ‘are’, ‘has been’, etc.) are used pervasively in scholarly writing, and while they are not wrong, they are boring. Why? Because little happens in sentences anchored by these verbs; no stance is taken, no movement occurs, no dramatic tension is introduced.

Second, the passive construction (‘has been discussed’) not only forces the use of the auxiliary verb ‘to be’, but also reduces energy by removing the agent of the action from the sentence. As Richard Lanham pointed out, you can’t tell ‘who’s kicking who?’ *(sic)* in a sentence in the passive voice [1]. Accordingly, in the example above you can’t tell who is doing the ‘discussing’.

Traditionally, the passive voice was required in scholarly writing because it conveyed a situation in which rigorous methods revealed facts but no person actually created them [2]. Many contemporary writers mistakenly believe that passive voice is a ‘rule’ they must obey. In many current scientific genres, however, active voice is not only allowed, it is preferred. It remains true that academic writing should have a balanced tone, but this does not mean that it has to be stagnant. The following revision increases the energy in this sentence through two strategies: it converts passive (‘has been discussed’) to active voice (‘have leveraged’) and changes a noun phrase (‘professionalism in medical students’) to an active verb (‘medical students develop and demonstrate their professionalism’):

*Medical education scholars* ***have leveraged*** *multiple perspectives* ***to understand*** *how medical students* ***develop and demonstrate*** *their professionalism.*

In this revision, the action is more robust—things are leveraged, developed and demonstrated—and it is being accomplished by clear agents—medical education scholars and medical students. It has energy, it carries the reader along.

To identify the passive voice in your sentences, ask yourself ‘who’s kicking who?’ If you can’t tell, you’re dealing with the passive. To revise into active voice, put the agent into the subject position (often the noun immediately prior to the verb). Consider this example:

*Our clerkship curriculum* ***was evaluated*** *using materials that* ***were prepared*** *to reflect the aims and challenges of workplace-based learning.*

Revision 1: ***We evaluated*** *the clerkship curriculum using materials* ***we prepared*** *to reflect the aims and challenges of workplace-based learning.*

In this revision, it is clear who is doing the actions of evaluating and preparing. However, if you are writing in a scientific culture that frowns on the use of the first person (in this case, *we*), you can still shift to active voice while avoiding the first person:

Revision 2: *Clerkship curriculum evaluation reflected the aims and challenges of workplace-based learning*.

This revision has made ‘clerkship curriculum evaluation’ into the agent of the new, active verb ‘reflected’.

In summary, when you convert passive to active voice and select energetic rather than limp verbs, your writing improves. But the take-home lesson is not that you must purge all passive voice and ‘to be’ verbs from your prose. (My first sentence in this manuscript used ‘to be’ as the main verb, and the sky didn’t fall.) Rather, the lesson is that the passive voice and limp verbs can produce sluggish academic prose, particularly if you use them pervasively and unconsciously. Now you are equipped to identify and work on these features, so that your writing takes full advantage of the power of the verb.
